# Characteristics of Disease Spectrum in relation to Species, Serogroups, and Adhesion Ability of Motile Aeromonads in Fish

**DOI:** 10.1100/2012/949358

**Published:** 2012-04-01

**Authors:** Alicja Kozińska, Agnieszka Pękala

**Affiliations:** Department of Fish Diseases, National Veterinary Research Institute, Al. Partyzantów 57, 24-100 Puławy, Poland

## Abstract

An attempt was made to delineate the relationship between of *Aeromonas* species and/or serogroups and specific disease symptoms in common carp *Cyprinus carpio* L. and rainbow trout *Oncorhynchus mykiss* Walbaum. The adhesion of *Aeromonas* strains to various tissues in relation to disease spectrum was also tested. All strains of *A. hydrophila* caused skin ulcers as well as septicaemia in both carp and trout while the other strains were able to cause only skin ulcers or some specific internal lesions with or without septicaemia depending on which species and/or serogroup they represented. Disease symptoms depended also on fish species. It was found that adhesion intensity of *Aeromonas* strains tested was significantly higher to tissues, which were susceptible to infection with these strains. The results indicate that adhesion to various cells of the fish organism is principal marker to detect virulent *Aeromonas* strains. The findings presented in this study may be helpful in the appraisal of aeromonads disease risk and kind of the infection in particular fish farms by epizootiological studies or/and during routine fish examinations. They will also be useful to improve and facilitate diagnosis of bacterial fish disease.

## 1. Introduction

The genus *Aeromonas* is composed of a large number of species. Currently, 17 genospecies and 14 phenospecies are recognized within this taxon [1]. Recently, seven newly described species have been proposed for inclusion to the genus *Aeromonas* [2]. The straight majority of species of the genus comprise motile and mesophilic strains and most of them are ubiquitous inhabitants of various aquatic ecosystems [3, 4]. *Aeromonas salmonicida* is only one species, which comprises nonmotile and psychrophilic strains. However, this species includes also motile strains referred to sometimes as *A. hydrophila*-like [1]. Motile and mesophilic *Aeromonas *spp. are well known as opportunistic but important pathogens of fish and other poikilothermic and homeothermic organisms including humans [2]. *A. hydrophila *and *A. veronii *bt. *sobria* are predominantly responsible for fish infections but *A. caviae*, *A. jandaei*, *A. sobria, A. bestiarum*, and mesophilic strains of *A. salmonicida* have also been reported as important pathogens of some fish species [5–8].

Mesophilic *Aeromonas* spp. show large serological diversity and include 96 established or provisional O-serogroups (SG) in NIH serotyping system (National Institute of Health, Japan) of Sakazaki and Shimada [9]. However, only some of them such as O3, O6, O11, O14, O16, O18, O21 O29 O33, and O41 seem to be associated with virulence for specific fish species [10–12].

Infections caused by mesophilic aeromonads in fish vary greatly in appearance. The pathological lesions may be only seen in the skin or internal organs but sometimes the lesions spread to other body sites causing systemic infection [6, 13–16]. It is commonly known that various stress factors and virulence level of *Aeromonas* strains have important influence on the disease severity. However, there are only partial data about the association of particular *Aeromonas* species and/or serogroups with disease spectrum in some fish species [6, 17, 18].

In the present study, an attempt was made to delineate the relationship between *Aeromonas* species and serogroups dominant in Polish fish farms and specific disease symptoms in common carp *Cyprinus carpio* L. and rainbow trout *Oncorhynchus mykiss* Walbaum. Moreover, the adhesion of the selected *Aeromonas *strains to skin, internal organs, and blood cells of these fish species was tested to determine the importance of this property in disease spectrum.

## 2. Material and Methods

### 2.1. Fish

Healthy common carp and rainbow trout weighing 80 to 100 g were used for the challenge tests. Fish used for adhesion tests were sampled at the moment when they grew up to the weight of 400 to 600 g. All fish originated from the same carp or trout farms, which were aeromonads-disease-free at least two years before the experiments. The fish were maintained in 300 l glass tanks with dechlorinated and aerated water before and during experiments. Water temperature was 20°C ± 1°C for carp and 12°C ± 1°C for trout. Fish were fed with pellets (Aller Aqua, Poland) suitable for the given fish species.

### 2.2. Bacterial Strains

*Aeromonas *strains were selected from those which have been previously identified to the species level, serogrouped, and classified as pathogenic for fish [7, 11]. All these strains showed similar pathogenicity factors, such as haemolytic and proteolytic activity, measured quantitatively as previously described [7, 19]. The strains were stored in trypticase soy broth (TSB) supplemented with 20% of glycerol at −80°C. The day before use, they were re-cultured on TSB and incubated overnight at 27°C.

### 2.3. Challenge

Fifty-one of *Aeromonas *strains were used for challenge. The strains represented the species *A. hydrophila*, *A. bestiarum*, *A. salmonicida* (mesophilic strains), *A. sobria*, and *A. veronii* bt. *sobria*, serogroups O3, O6, O11, O16, O18, O21, O29, O33, O41, and six provisional groups O (PGO) (Table 1). The 24 h bacterial cultures in TSB were diluted in sterile phosphate buffered saline (PBS) to the final concentration of 5 × 10^6^ bacterial cells mL^−1^. Before infection, fish were anaesthetized by bath for 2–5 min. in solution of MS-222 (Sigma), at the concentration from 75 to 150 *μ*g L^−1^ of water (lower doses for trouts and higher for carps). For each strain, five carps and five trouts were injected subcutaneously (Sc) with 0.1 mL of the diluted bacterial culture. The same numbers of other individuals were injected intraperitoneally (Ip) with 0.5 mL of the same inoculum. Symptoms of the disease were recorded daily during two weeks. The fish being in death throes or freshly dead were used for clinical, postmortem, and bacteriological examinations. Skin, liver, kidney, and blood samples were taken for bacteriological tests.

Local Ethic Commission in Lublin approved the procedure concerning experiments on fish.

### 2.4. Adhesion to Skin, Internal Organs, and Blood Cells

Selected 20 *Aeromonas* strains causing different disease symptoms were used for these tests (see Table 4). The strains were grown overnight in TSB at 27°C ± 1°C and centrifuged at 3,000 g for 10 min, and the bacterial pellets were suspended in PBS to final concentration of 10^7^ cells mL^−1^. Fish were killed by bath in the suspension of MS-222 at the lethal concentration for 10–15 min. Then they were washed under tap water and disinfected in 70% ethanol. One square centimeter of carp and trout skin (CS and TS, resp.), and 0.5 g of carp kidney (CK) and trout liver (TL) were taken. The samples were placed in separate Petri dishes containing 30 mL of particular strain suspensions and incubated for 1 h at room temperature with continuous gentle shaking. Then, the samples were carefully washed by dipping five times in containers with sterile PBS and finally under stream of the saline. After homogenization, several tenfold dilutions were performed and 100 *μ*L of the material from each dilution was inoculated onto blood agar (BA) and incubated for 48 h at 27°C ± 1°C. Colonies were counted, and, after considering the dilution, the number of colony-forming units (cfus) was determined. The samples of CS, TS, CK, and TL exposed to sterile PBS were used as controls. For photographic documentation, adhesion was also tested on microscopic slides. After washing and disinfection of fish, surface mucous with epidermis, CK and TL were taken separately, diluted in sterile PBS in the ratio of 1 : 2 and homogenized. Additionally, carp and trout blood was also used. One hundred microliters of mucous, CK and TL homogenates and three drops of blood were smeared onto slides, air-dried, and fixed for 10 min with methanol. Then, the slides were incubated as described above and washed under quite strong stream of sterile PBS. The slides were stained by the Gram method and examined under a light microscope. Controls were incubated in sterile PBS. The photographs were made by camera connected with the microscope.

### 2.5. Statistical Analysis

Adhesion intensity (number of cfus) of two groups of *Aeromonas* strains was compared. The first one contains all strains signed as “+” and the second one contains all strains signed as “−” (see Table 5). At first, F-Seconder's test was used to check if variances of the two groups are statistically consistent and then U-Mann-Whitney-Wilcoxon's test was used to compare means of the data of the two groups at *α* ≤ 0.05.

## 3. Results

### 3.1. Disease Symptoms after Challenge in relation to *Aeromonas* Species and Serogroups

**Figure 1:**
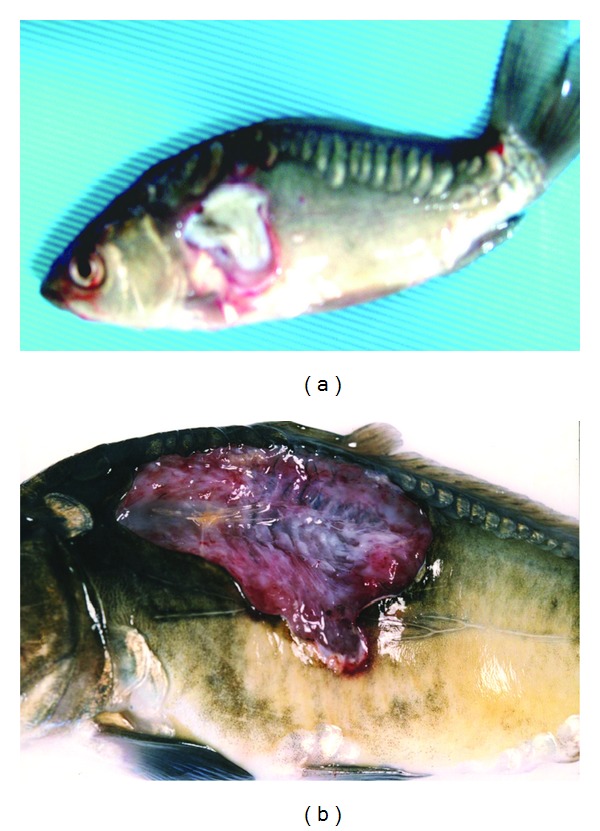
Ulcers in carps Sc challenged with *A. hydrophila *strain Pt39 (a) and *A. bestiarum* strain J4N (b). Very deep ulcer exposing skeleton after injection of *A. bestiarum*.

**Figure 2:**
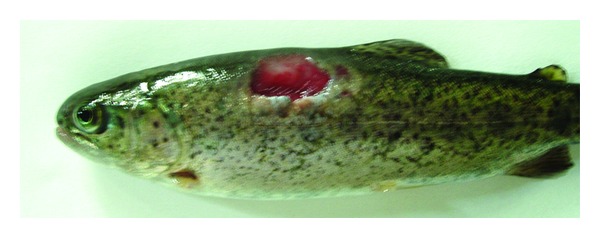
Skin ulcer penetrating deep into the muscle in trout Sc challenged with *A. hydrophila* Pt104.

**Figure 3:**
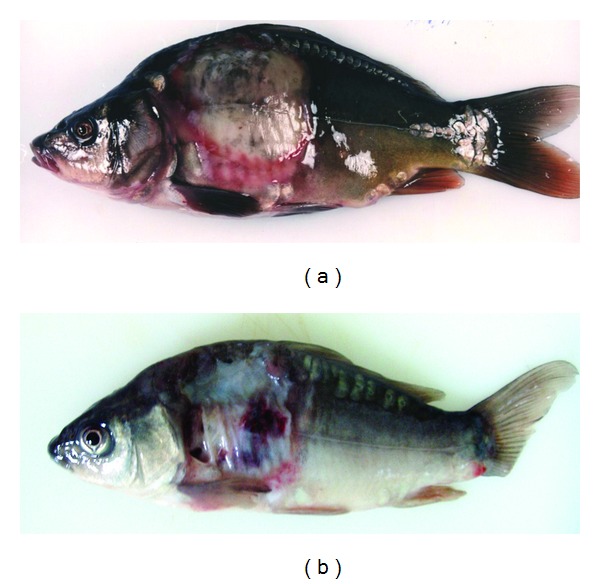
Extensive dermatitis in carp Sc challenged with *A. veronii *bt. *sobria* strains Pt 57 (a) and K48 (b). Distended anus (arrow) was the first symptom of septicaemia.

**Figure 4:**
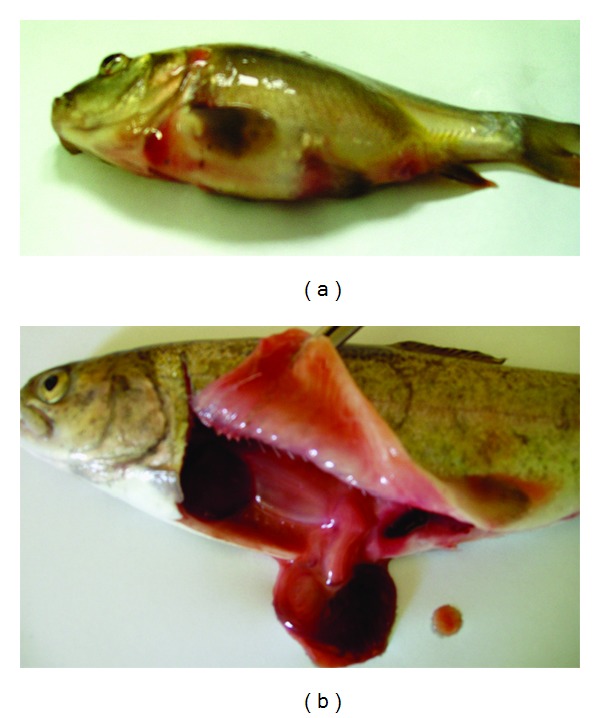
Septicaemia in fish Ip challenged with *A. veronii bt. sobria *strain K151 (a) and *A. hydrophila* strain Pt104 (b): exophthalmia, swelling of abdomen, and intensive congestions on the body surface in carp (a); enlarged spleen, intensive congestions, and liquefaction of internal organs in trout (b).

The ability of particular *Aeromonas* strains to cause specific disease symptoms after challenge of carp and trout are presented in Tables 2 and 3, respectively, and in Figures 1, 2, 3, and 4.

All strains except one of *A. veronii* bt. *sobria* (serogroup O41) caused external lesions on the body surface in carps after Sc challenge. Skin ulcers penetrating into subcutaneous muscle were usually formed (Figure 1). Skin ulcers in trout were formed only after Sc injection with all strains of *A. hydrophila* (Figure 2) and *A. veronii* bt. *sobria* except one belonging to serogroup O41. Similar lesions in trout were also caused by *A. bestiarum* SGs O16, PGO2, and PGO6 and *A. salmonicida* SG O3 strains. *A. veronii* bt. *sobria *strains belonging to serogroups O6 and O11 caused especially extensive dermatitis in carp (Figure 3) and symptoms of septicaemia such as distended anus abdomen swelling, exophthalmia, ascitic fluid in peritoneal cavity, anaemia or haemorrhages in internal organs, kidney watery, and jelly-like discharge in the intestine were observed. All fish died within 2–4 days after challenge. The remaining strains did not cause lesions in internal organs after Sc challenge.

Ip challenge with each strain of *A. hydrophila* resulted in septicaemia in both carp and trout with the symptoms described above. Similar symptoms were caused by all strains of *A. veronii* bt. *sobria* (Figure 4(a)) and *A. salmonicida* belonging to SG O3 in carp and by all strains of *A. sobria* and *A. salmonicida* (Figure 4(b)) except one strain (A16) of the later species in trout. The disease showed acute form and 60% to 100% of infected fish died during 4 to 7 days after challenge. The strains belonging to the remaining *Aeromonas* species or serogroups caused relatively mild lesions such as increased moistness, anaemia, sometimes haemorrhages in internal organs and enlargement spleen or did cause no disease symptom after Ip injection (Tables 2 and 3).

Bacteria used to challenge were reisolated in all cases from the affected tissues and also from blood when systemic infection observed.

### 3.2. Disease Symptoms in relation to Adhesion Ability

**Figure 5:**
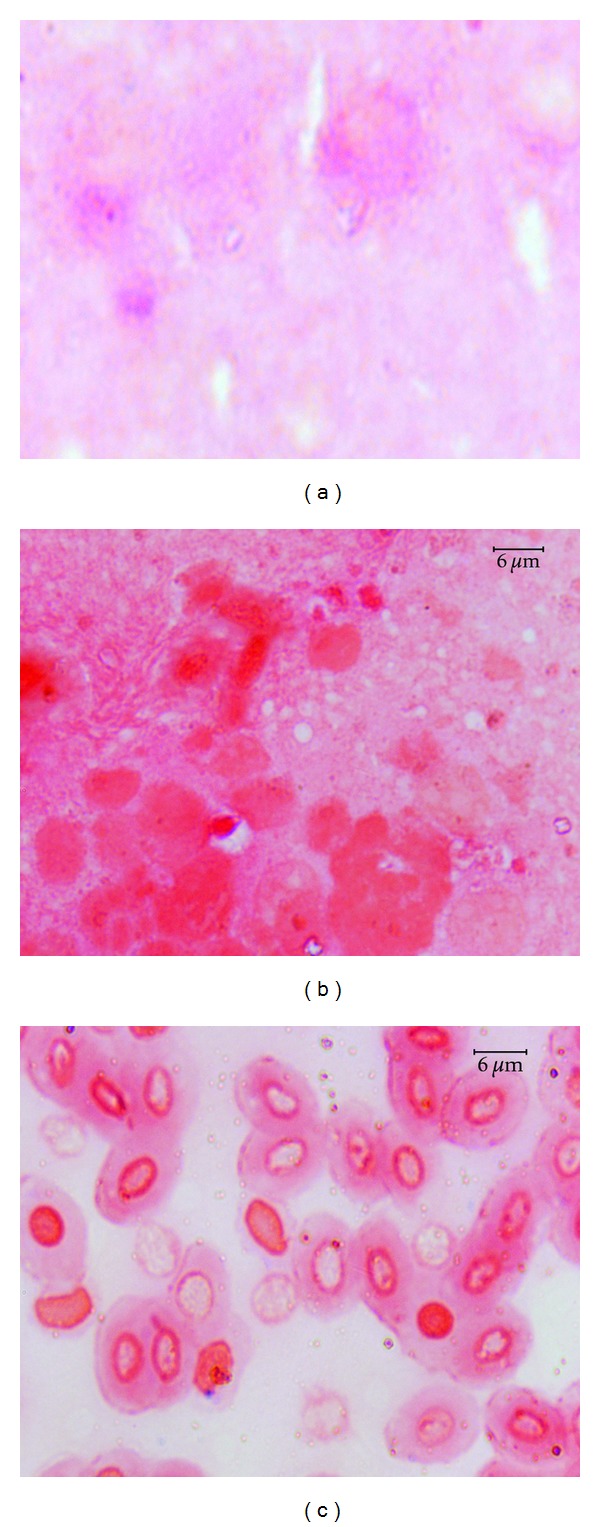
Control slides covered with homogenized carp skin mucus (a), trout liver (b), and carp blood (c): no bacterial cells are visible.

**Figure 6:**
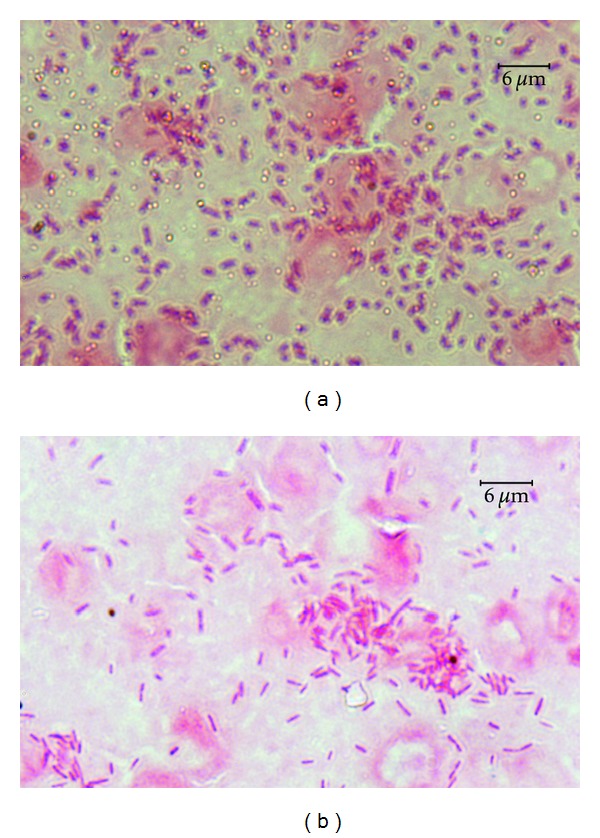
Numerous bacterial cells of *A. veronii* bt. *sobria* strain K48 adhering to carp mucus and epidermal cells (a) and carp kidney (b).

**Figure 7:**
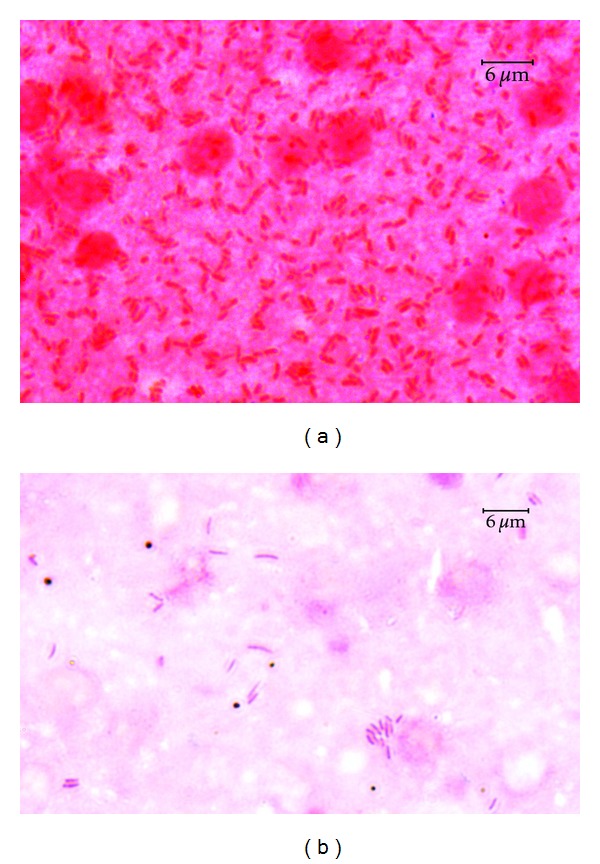
Numerous bacterial cells of *A. hydrophila *strain Pt104 (a) adhering to trout liver (a) and isolated bacterial cells of *A. sobria* strain K24 adhering to carp kidney (b).

**Figure 8:**
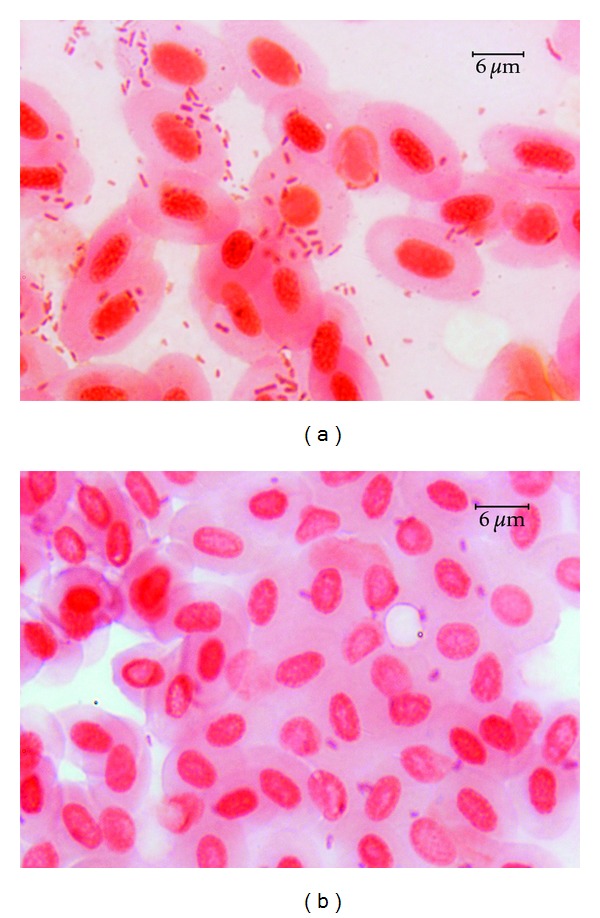
Numerous bacterial cells of *A. hydrophila* strain Pt 104 (a) and isolated cells of *A. veronii* bt. *sobria* strain W62 (b) adhering to trout blood cells.

No bacteria were detected in the control samples of the skin and internal organs after incubation on either BA or coated slides (Figure 5). The cfu numbers from 5 × 10^2^ to 6.8 × 10^7^ were received from the samples exposed to particular *Aeromonas* strains (Table 4). All strains causing skin ulcers, dermatitis, or any lesions in internal organs in the given fish species showed strong adhesion to these tissues at mean number >10^6^ cfu 1 square cm^−1^ of the skin and >10^7^ cfu 0.5 g^−1^ of internal organs (Table 5 and Figures 6 and 7(a)). Similar level of adhesion to carp skin and trout liver was noted only for two strains (W62 and K48, resp.) which did not cause disease symptom in these tissues (Table 4). The remaining strains from the groups unable to cause external or internal lesions in particular fish species adhered poorly to skin or internal organs (Figure 7(b)). The means of cfu 1square cm^−1^ of the skin or 0.5 g^−1^ of internal organs amounted to 4.2 × 10^3^ or 6.3 × 10^3^, respectively (Table 5). Numerous bacterial cells were visible on the slides covered with blood and exposed to the strains, which were able to cause septicaemia (Figure 8(a)) while the remaining strains adhered to blood cells very poorly (Figure 8(b)) and the majority of them were unnoticeable on the slides at all.

#### 3.2.1. Statistical Analysis

The ratio of variances for the two group data was *F* = 0.2023, *P* value = 1.109e-06, so these data were not consistent. Using U-Mann-Whitney-Wilcoxon's test, the results were *W* = 1352.5, *P* value = 5.222e-12. Zero hypothesis (conformity of the data) was rejected on the basis *P* value. Therefore, the difference between means of the two groups were statistically significant at *α* ≤ 0.05.

## 4. Discussion

Mesophilic aeromonads are a peculiar group of bacteria because of their large taxonomic and serological diversity and at the same time *Aeromonas *serogroups are not species specific [11, 20, 21]. According to Popoff's [22] classifications of the genus *Aeromonas*, *A. hydrophila*, *A. caviae*, and *A. sobria* have been reported as the species responsible for the most different conditions in fish. It is currently known that each of these species contains 3–5 hybridization groups, and at least 14 phenotypically described separate *Aeromonas* species are recognized [1]. This fact as well as serological diversity of motile aeromonads suggested the relationship may appear between specific disease symptoms and currently recognized species or/and serogroups. It has been, at some degree, shown for *Aeromonas* strains isolated from various human specimens [2, 21, 23, 24].

This is the first study on the determination of similar links in fish infections caused by motile aeromonads. It is very important problem because a *Aeromonas* spp. constitute very often the component of mixed bacterial flora isolated from asymptomatic carriers as well as from fish with various disease conditions caused sometimes by bacteria belonging to completely different taxa. In such cases the correct diagnosis is very difficult.

In this study, all carps and all trouts originated from the same populations and remained under identical conditions, suitable for particular fish species, during experiments. Furthermore, identical doses of each strain were used for challenge tests. All these factors gave good comparability and reliability of the results.

No relationship between *Aeromonas* spp. or SG and their ability to cause lesions on the carps skin was observed. All strains except one formed less or more extensive ulcers or dermatitis. However, skin ulcers in trout were only caused by the strains belonging to some *Aeromonas* species or/and serogroups. The presence of any lesions in internal organs with or without septicaemia syndrome in both carp and trout depended also markedly on taxonomic membership of the strains. At the same time, it is worth stressing that specific disease symptoms caused by strains belonging to all species except *A. hydrophila* varied also depending on fish species. All strains of *A. veronii* bt. *sobria* caused septicaemia only in carp. The species has also been described as the causative agent of septicaemia syndrome in spotted sand bas [17]. From 8 serogroups of *A. salmonicida* used to challenge, only strains belonging to SG O3 were able to cause systemic infection in carp. In contrast, septicaemia syndrome in trout was observed after challenge with all strains of *A. salmonicida*, and *A. sobria*. The latter species has been described as a causative agent of serious disease in perch [8]. No *A. bestiarum* strain was able to cause septicaemia symptoms although all caused relatively mild lesions in internal organs of trout. Only some strains (SGs O3 and O11) of the species caused similar lesions in carp and some others (SGs O16, PGO2 and PGO6) skin ulcers in trout. *A. hydrophila* was only one species able to cause ulcers as well as systemic infection in both carp and trout. This species has also been reported as one of *Aeromonas* spp. responsible for haemorrhaging septicaemia in eels [6]. It should be stressed that *A. hydrophila* and *A. veronii* bt. *sobria,* especially from SGs O11 and O16, are also predominant in human blood-borne infections and the latter species also in gastroenteritis [2, 21, 24, 25]. It indicates that fish infected with these species may be hazardous for human health.

Pathogenicity of motile aeromonads is multifactorial, but adhesion and colonization of various host cells seem to be the most important factor for initiation of disease process [2, 26–28]. However, some investigators have found no correlation between virulence and adhesion ability of motile aeromonads [29]. All *Aeromonas* strains tested in this study were virulent for fish as has previously been showed [7, 11]. However, most of these strains, except *A. hydrophila*, caused disparate disease symptoms depending on *Aeromonas *species and/or serogroups. It may be associated with the kind of bacterial adhesins. Structures such as fimbria (pili), LPS, outer membranes (OmpA), and lectins have been reported as adhesins of *Aeromonas* spp. [17, 26, 30–32]. Possibly, these adhesive factors are *Aeromonas* species specific. There has been shown that adhesion factors of *A. veronii* are cell-associated lectin MCBP or OmpA [17, 32], whereas structures such as pili, LPS and OmpII were reported as adhesins in *A. hydrophila* [26, 31, 33]. Various adhesin structures may explain the ability of the strains belonging to these species, especially *A. hydrophila,* to cause large spectrum, of disease in various fish species.

Bacterial adhesion processes involve also specific receptor structures on fish cells such as glycoproteins of intestinal gut mucus [28] or mucin, lactoferrin, and collagen [17]. Carbohydrate-rich substrates have also been suggested as receptors for *Aeromonas* adhesins [34]. Therefore, various models such as trout and carp skin and blood, carp kidney, and trout liver were used for adhesion tests in this study. It was found very good correlation between adhesion intensity to particular tissues and their susceptibility to infection. Adhesion intensity of particular *Aeromonas *strains to tissues susceptible to infection was significantly higher than to those which were not affected by them. This indicates that specific receptor structures for *Aeromonas *adhesins are different on the skin mucus, internal organs, and blood cells. They seem also to be fish species specific. This confirms the previous suggestions that fish skin mucus has host-specific properties [27].

Carp skin was susceptible to infection of all *Aeromonas *strains used to challenge and at the same time all strains tested for adhesion showed intensive adhesion ability to the tissue. Probably, in carp body surface mucus are located receptors specific for various adhesive factors of particular *Aeromonas* species and serogroups.

 The results of this study indicate that the presence of *Aeromonas* bacteria in fish tissue samples is not necessarily a sign that they can cause disease or are causative agent of observed disorders. The presented findings provided evidence that even bacteria commonly known as pathogenic for fish are not able to cause pathological symptoms in some body sites if their adhesion is very poor to specific tissues. Therefore, isolation of such bacteria from mixed bacterial flora does not always indicate that they are primary factor of a disease. It should be stressed that *Aeromonas* bacteria overgrow often some other pathogenic microorganisms, for example, *A. salmonicida* ssp. *salmonicida* or *Flavobacterium *spp [5], which require longer time to grow. In such cases isolation of these bacteria is very difficult. As was shown in this study, *Aeromonas* strains unable to cause lesions adhered weakly to tissue and were easily removed from it by washing. This fact may be of use during preparation of fish tissue material. Our preliminary comparative studies showed that the washing of the skin, gills, and some internal organs (with tight consistency) markedly reduced *Aeromonas *cells and the proportion of these bacteria to other microorganisms markedly decreased. The picture of bacterial culture was then more clear and facilitated isolation of pathogens such as *Aeromonas salmonicida* ssp. *salmonicida*, *Flavobacterium columnare,* and *Streptococcus* spp. (data not published).

## 5. Conclusions

From the data presented in this paper, it is apparent that *A. hydrophila* is the most versatile and dangerous species among fish pathogens from the genus *Aeromonas*. Probably it is associated with the presence of various types of adhesins. Moreover, *A. veronii* bt. *sobria* was found as especially dangerous pathogen for carps and *A. salmonicida* and *A. sobria* for trouts. All these species are able to cause acute form of disease with septicaemia syndrome. There was found that disease symptoms caused by *Aeromonas* spp., except *A. hydrophila*, are specific for both bacteria and fish species to a considerable degree. There seems to be poor correlation between *Aeromonas* serogroups and the disease picture although it was found among the strains belonging to the species *A. bestiarum*, *A. salmonicida*, and *A. veronii bt. sobria*. There was evident correlation between adhesion intensity of the strains to specific fish tissues and disease spectrum caused by them. This indicates that adhesion to various cells of fish organism may be the principal marker to detect virulent *Aeromonas* strains, which may cause specific disease spectrum in carps or/and trouts.

The findings presented in this study, especially concerning different ability of particular *Aeromonas* species and some serogroups of these bacteria to cause specific lesions in carp and trout, may be helpful in an appraisal of aeromonad disease risk and the kind of the infection in particular fish farms by epizootiological studies or/and during routine fish examinations. Adhesion ability may facilitate and improve diagnosis of bacterial fish diseases. However, adhesion of *Aeromonas* spp. may be mediated by both specific and nonspecific charge and hydrophobic interactions [17]. Therefore, complementary studies are needed in order to better understand the type of adhesion process of particular *Aeromonas* species and serogroups to various fish tissues.

## Figures and Tables

**Table 1:** *Aeromonas *strains used for challenge tests.

* Aeromonas* species	Strains	Serogroup
*A. hydrophila*	Pt39, Pt40	O11
	Pt104, Pt109	O16
	W58	PGO10
	W68	PGO11

*A. bestiarum*	J4N, K15S	O3
	K167, K206	O11
	P1W, K2	O16
	K14S, K296	O18
	K190, Pt303	O33
	K301, K333	PGO1
	Pt16	PGO2
	K339	PGO4
	P1S	PGO6

*A. salmonicida*	K401, K402	O3
	W32	O11
	K299, K299B	O16
	K352A, K352C	O33
	A16	O18
	1S91	PGO1
	A11, A17	PGO2
	1S95, K116	PGO6

*A. sobria*	K24, K24C	O3
	K150, K354	O16
	K311	PGO1

*A. veronii *bt*. sobria *	S4W, K48, K348	O6
	K144, Pt57	O11
	K151, K202	O33
	W62, K166, K170	O41
	K156	PGO2
	K168	PGO4

**Table 2:** Disease symptoms in challenged carp in relation to *Aeromonas* species and serogroups.

Lesions after challenge	Species	Serogroups
Only external (ulcers or dermatitis)	*A. bestiarum*	O16, O18, O33, PGO1, PGO2, PGO4
	*A. salmonicida*	O11, O16, O33, O18, PGO1, PGO2, PGO6
	*A. sobria*	O3, O16, PGO1
Only internal	*A. veronii *bt. sobria**	O41 (strain W62)
Both external and internal	A. hydrophila**	O11, O16, PGO10, PGO11
	A. bestiarum*	O3, O11, PGO6
	A. salmonicida**	O3
	*A. veronii *bt. sobria**	O6, O11, O33, O41 (strains K166, K170), PGO2, PGO4

* Increased moistness of internal organs, enlarged spleen, anaemia and/or haemorrhages in liver-pancreas; **systemic infection.

**Table 3:** Disease symptoms in challenged trout in relation to *Aeromonas *species and serogroups.

Lesions after challenge	*Aeromonas *species	*Aeromonas* serogroups
Only external (ulcers)	*A. veronii *bt*. sobria *	O6, O41 (strains K166, K170), PGO2, PGO4
Only internal	A. bestiarum*	O3, O11, O33, PGO1, PGO4
	A. salmonicida**	O11, O16, O33, PGO1, PGO2, PGO6
	A. sobria**	O3, O16, PGO1
Both external and internal	A. hydrophila**	O11, O16, PGO10, PGO11
	A. bestiarum*	O16, PGO2, PGO6
	A. salmonicida**	O11, O16, O33, PGO1, PGO2, PGO6
	*A. veronii* bt. sobria*	O11, O33

* Increased moistness, anaemia and/or haemorrhages in internal organs, enlarged spleen; **systemic infection.

**Table 4:** The ability of *Aeromonas* strains to cause pathological lesions in carp and trout skin and internal organs in relation to their adhesion to these tissues.

Strain	Ability to cause lesions in tissues				No. of bacterial cells adhered to tissues			
	CS	CIO	TS	TIO	CS	CK	TS	TL
Pt39	+	+	+	+	2.2 × 10^6^	6 × 10^6^	3.5 × 10^6^	10^7^
Pt104	+	+	+	+	8.5 × 10^6^	4.7 × 10^6^	3.8 × 10^6^	8 × 10^6^
J4N	+	+	−	+	3.6 × 10^6^	5.9 × 10^6^	5.5 × 10^2^	4.5 × 10^6^
K167	+	+	−	+	4 × 10^6^	5 × 10^6^	2 × 10^3^	2.6 × 10^6^
K206	+	+	−	+	5.2 × 10^6^	8.2 × 10^6^	10^4^	7 × 10^6^
K2	+	−	+	+	6 × 10^6^	4 × 10^3^	5 × 10^6^	6 × 10^6^
P1W	+	−	+	+	9.6 × 10^5^	4.5 × 10^3^	9.5 × 10^5^	2.2 × 10^7^
Pt303	+	−	−	+	10^6^	2 × 10^3^	9 × 10^3^	8.8 × 10^6^
K402	+	+	+	+	2.6 × 10^6^	2 × 10^6^	4.8 × 10^6^	10^7^
K299	+	−	−	+	3.6 × 10^6^	6 × 10^4^	1.3 × 10^4^	2 × 10^7^
A16	+	−	−	−	9.2 × 10^5^	6 × 10^2^	2 × 10^3^	6 × 10^2^
K352A	+	−	−	+	2.9 × 10^6^	2.7 × 10^4^	5 × 10^2^	3 × 10^7^
K24	+	−	−	+	1.2 × 10^6^	3 × 10^4^	3.5 × 10^3^	6.8 × 10^7^
K354	+	−	−	+	9.5 × 10^5^	8 × 10^2^	6 × 10^2^	7.3 × 10^6^
K48	+	+	+	−	2 × 10^7^	6 × 10^7^	9 × 10^6^	2.8 × 10^6^
Pt57	+	+	+	+	2.9 × 10^7^	5.3 × 10^7^	6.8 × 10^6^	6.5 × 10^6^
K1170	+	+	+	−	4 × 10^6^	6 × 10^6^	8.5 × 10^6^	2.4 × 10^4^
W62	−	+	−	−	2.5 × 10^6^	6 × 10^6^	5 × 10^2^	2 × 10^3^
K166	+	+	+	−	9 × 10^6^	9 × 10^6^	6.6 × 10^6^	4.2 × 10^3^
K168	+	+	+	−	2.8 × 10^6^	9.5 × 10^6^	8.8 × 10^5^	6.5 × 10^2^

CS and TS: carp and trout skin, respectively; CIO and TIO: carp and trout internal organs, respectively; CK: carp kidney; TL: trout liver.

**Table 5:** Adhesion intensity of groups of *Aeromonas* strains which were able (+) or unable (−) to cause skin ulcers or dermatitis (SU/D) or any lesions in internal organs (IOL) in both carp and trout.

* Aeromonas* strains		Mean number of bacterial cells adhered to skin and internal organs
Group	Number	
SU/D+	29	5.2 × 10^6^
SU/D−	11	2.3 × 10^5^(4.2 × 10^3^)*
IOL+	26	1.2 × 10^7^
IOL−	14	2.1 × 10^5^ (6.3 × 10^3^)*

*Data after rejection extreme results for the strains W62 (group SU/D−) and K48 (group IOL−).
